# Comprehensive genomic analysis of hypocholesterolemic probiotic *Enterococcus faecium* LR13 reveals unique proteins involved in cholesterol-assimilation

**DOI:** 10.3389/fnut.2023.1082566

**Published:** 2023-04-04

**Authors:** Manisha Aswal, Neelja Singhal, Manish Kumar

**Affiliations:** Department of Biophysics, University of Delhi South Campus, New Delhi, India

**Keywords:** cholesterol-assimilation, mobile genetic elements, probiotics, metabolic effect, hypercholesterolemia

## Abstract

Hypercholesterolemia is a major risk factor for cardiovascular diseases (CVDs). Chemotherapeutic agents for CVDs exhibit several side effects. Specific probiotics with hypocholesterolemic effects can be safe and effective alternatives to chemotherapeutics. Here, we have analyzed and compared the genome of a novel rhizospheric *Enterococcus faecium* LR13 cholesterol-assimilating probiotic with other probiotic/pathogenic *E. faecium* strains to discern genetic factors underlying probiotic efficacy and cholesterol-assimilation. Genomic analyses of *E. faecium* probiotic strains revealed that LR13 and WEFA23 (cholesterol-assimilating probiotics) harbored 21 unique proteins absent in non-cholesterol-assimilating probiotics. Of these, 14 proteins could directly help in cholesterol-assimilation by producing short chain fatty acids, lipid (sterol) transport and membrane stabilization, and bile salt hydrolase activity. This suggests that cholesterol-assimilation is an intrinsic, strain-specific trait exhibited by probiotics with a specific genetic constitution. Moreover, the unique proteins identified in this study can serve as biomarkers for discerning/characterizing cholesterol-assimilating probiotics as novel biotherapeutics against CVDs.

## Graphical Abstract

The three probable mechanisms by which the unique proteins discerned in hypo-cholesterolemic *E. faecium* LR13 and WEFA23 probiotic strains might aid in cholesterol-assimilation : (i) *bile salt hydrolysis*: bacterial bile salt hydrolases (BshB) convert taurine/glycine cholic acid (T/GCA) to cholic acid (CA) that aids in the activation of cholesterol 7α-hydroxylase (Cyp7a) in the liver resulting in degradation of cholesterol into bile acids (110), (ii) *production of short chain fatty acids (SCFAs)*: the bacterial proteins YebG, TesA, ENGase, BglG, DdcY, and UPI000C77AD8E are involved in the uptake and breakdown of dietary fibers into short chain fatty acids (SCFAs) like propionate, acetate and butyrate. In the liver, these inhibit HMG-CoA reductase enzyme (3-hydroxy-3-methylglutaryl-CoA) which converts HMG-CoA to mevalonic acid resulting in inhibition of cholesterol biosynthesis, and (iii) *lipid (sterol) transport, membrane stabilization and binding of cholesterol to the bacterial cell walls*: bacterial proteins Q3Y02, Q9KRT5, P7903, U2Q622, U2NW17, D5HKK5, and P9WK41 aid in uptake of sterols (lipids) from the intestinal lumen and incorporation in their cell membranes/walls. Created by BioRender.com. 
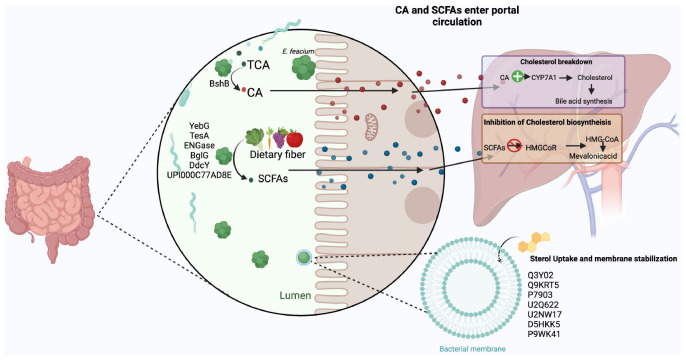

## Background

Elevated serum cholesterol is a major risk factor for cardiovascular diseases (CVDs). Though statins and other drugs are effective in lowering serum cholesterol, they have several side effects. Thus, agents which can exhibit hypocholesterolemic effects without side effects should be discovered. Recently, probiotics have been promoted as important food additives/supplements for improving human health. According to the World Health Organization (WHO) and Food and Agricultural Organization (WHO/FAO) probiotics may be defined as, “live microbes which when administered in a certain amount deliver several health benefits on the host” (1). Probiotics are mainly associated with the maintenance of a healthy gastrointestinal tract (GIT) by restoring the microbial balance of the host gut (2). However, several studies have reported that probiotics might also confer some other health benefits, like prevention of oral/mouth diseases, bacterial/fungal vaginal infections, cancer, osteoporosis, type 2 diabetes, CVDs, antibiotics induced diarrhea, irritable bowel syndrome (IBS), etc. (2, 3). Various researchers have identified many species/strains of the genera *Lactobacillus, Lactococcus, Levilactobacillus*, *Limosilactobacillus*, *Pediococcus*, *Streptococcus*, *Weissella*, *Enterococcus*, etc. with probiotic potential and/or other health benefits (4).

*Enterococcus faecium* is a common commensal of the human GIT. *E. faecium* species is composed of both probiotic and pathogenic strains and reportedly due to genomic diversity and plasticity, *E. faecium* strains might exhibit probiotic or pathogenic traits (5–7). Some pathogenic strains of *E. faecium* carrying virulence and antibiotic resistance genes, especially vancomycin-resistant genes, were reported to be responsible for severe nosocomial outbreaks (8, 9). These *E. faecium* strains were known as vancomycin-resistant enterococci (VRE). Also, these *E. faecium* (VRE) strains were categorized in the WHO “Global Priority List of Antibiotic-resistant Bacteria to Guide Research, Discovery and Development of New Antibiotics” under priority 2 (high) category (10). On the other hand, many *E. faecium* strains were reportedly associated with health benefits such as regulation of gut microbiota, reduction of IBS symptoms, allergy and diarrhea (2, 11), reduction of obesity, immune system modulation (12), lowering of cholesterol-levels (13), etc. Many strains of *E. faecium*, *E. faecium* T110 (14), *E. faecium* 170M39 (15), *E. faecium* SP15 (16), *E. faecium* WEFA23 (17), *E. faecium* LR13 (18) have been reported as potential probiotics and, *E. faecium* SF68^®^, *Enterococcus faecalis* Symbioflor^®^ 1, etc. (11, 19) are approved commercial probiotics. Reportedly, specific genomic differences were found to exist in the food-grade and nosocomial enterococci strains (20).

Most of the current probiotics available in Indian markets are non-indigenous strains whose efficacy is dubious (21, 22). Due to different genetic compositions and geographical variations, the microbiome of the Indian population is different from the microbiomes of the populations living in other parts of the world (23, 24). Indigenous probiotic strains isolated from animal or environmental niches specific to a particular geographical location might help in identifying population-specific probiotics which can confer better health benefits on the local population than non-indigenous strains.

In an earlier study we had reported a rhizospheric isolate of *E. faecium* LR13 which exhibited several probiotic attributes and hypo-cholesterolemic potential *in vitro* (18). Also, this strain was found to be devoid of virulence factors and antibiotic resistance genes which suggested its use as a potential probiotic candidate. In the present study we have performed *de novo* complete genome analysis of the indigenous *E. faecium* LR13 strain to unravel the genes/pathways associated with the probiotic traits. Since, mobile genetic elements (MGEs) such as plasmids, insertion sequences, prophages, pathogenicity islands, and transposons are reportedly involved in transmission of traits such as virulence, antimicrobial resistance (AMR), and probiotic attributes (25–29), MGEs were also characterized to understand if the AMR/virulence/probiotic traits depicted by *E. faecium* LR13 were intrinsic or acquired. Additionally, the genome sequence of *E. faecium* LR13 was compared with the genome sequences of other probiotic strains such as, *E. faecium* T110 (14), *E. faecium* 170M39 (15), *E. faecium* SP15 (16), and *E. faecium* WEFA23 (17) to understand its genomic relatedness with *E. faecium* probiotic strains isolated from other parts of the world. Also, the genome of *E. faecium* LR13 was compared with the genomes of pathogenic and non-pathogenic non-probiotic *E. faecium* strains to determine its genomic relatedness/differences with other *E. faecium* strains.

## Materials and methods

### Revival of *E. faecium* LR13 and DNA isolation

*Enterococcus faecium* LR13 preserved as glycerol stock (50% v/v) at −80°C in our laboratory was revived by incubating overnight in deMan, Rogosa and Sharpe (MRS) broth at 37°C, 200 rpm. Bacteria were grown up to the exponential phase (OD_600_ = 0.8) and harvested by centrifugation at 8,000 rpm for 8 min at 4°C. Genomic DNA was extracted using Nucleospin Microbial DNA kit (Takara Bio, San Jose, CA, USA) following the manufacturer’s instructions. DNA concentrations were determined using Qubit 4 fluorometer (Thermo Fisher Scientific, Waltham, MA, USA).

### Genome sequencing and assembly

The sequencing DNA library was prepared by QIASeq FX DNA Library Kit (Qiagen, USA). Quantitative and qualitative library QC was done by Qubit 4 fluorometer (Thermo Fisher Scientific, Waltham, MA, USA) and tapestation 4150 (Agilent technologies, Santa Clara, CA, USA), respectively. The libraries were sequenced on Novaseq 6000 (Illumina, USA) by using 2 × 150 bp paired end sequencing chemistry. The quality of the raw reads was assessed using FastQC v0.11.8 (30) and BLAST against NCBI Nucleotide Database (NT) at *e*-value 1e−6. The reads were trimmed using Trimmomatic v0.39 (31) at default parameters to remove Illumina adaptors, low-quality bases and/or reads less than 36 bp. The trimmed short overlapped paired-end reads were merged using Flash (v1.2.11) (32) at default parameters to create longer reads (single-end). The merged single-end reads along with the remaining trimmed pair-end reads were used to perform *de novo* genome assembly using SPAdes v3.13.0 (33) and Unicycler v0.4.8 (34). From the assembled contigs, all contigs with ≤200 bp length were discarded using SeqKit v0.16.1 (35). The quality of the assembled genome was further assessed using QUAST v5.0.2 (36) and checkM v1.1.3 (37).

### Genome annotation

The assembled genome was annotated using prokaryotic genome annotation pipeline PGAP (2021-07-01) (38), Prokka v1.14.6 (39), KAAS server with BBH (bi-directional best hit) (40) and PATRIC RAST tool kit (RASTtk) v3.6.12 (41). The protein coding genes, tRNA and rRNA genes were predicted using Glimmer version v3.02 (42), tRNAscan-SE v2.0.9 (43), and RNAmmer v1.2 (44), respectively.

### Essential and desirable probiotic genes

The presence of essential probiotic genes in *E. faecium* LR13 was confirmed by ascertaining the presence of genes essential for survival in the human gut such as *dltA* (D-alanine D-alanyl carrier protein ligase), *dltB* (teichoic acid D-alanyltransferase), *copA* (copper-importing P-type ATPase A), *gadC* (glutamate/gamma-aminobutyrate antiporter), *dps* (DNA protection during starvation), *clpC* (ATP-dependent Clp protease ATP-binding subunit ClpC), *clpE* (ATP-dependent Clp protease ATP-binding subunit ClpE), *msrB* (peptide methionine sulfoxide reductase), *treC* (tellurium resistance protein), and *bsh* (bile salt hydrolase) (14).

Presence of genes for desirable probiotic attributes such as biosynthesis of essential amino acids and vitamins was determined by complete genome classification of *E. faecium* LR13 using the PANTHER database (45) and mapping of biosynthetic pathways on the KEGG database (46). The presence of phosphoenolpyruvate-dependent sugar phosphotransferase (sugar PTS) genes such as, *ptsG_1* (PTS system glucose-specific EIICB component), *ptsG_2* (PTS system glucose-specific EIICB component), *ptsG_3* (PTS system glucose-specific EIICB component), *ptsH* (phosphocarrier protein HPr), *ptsI* (phosphoenolpyruvate protein phosphotransferase), and *ptsP* (phosphoenolpyruvate protein phosphotransferase) (47) and biofilm formation such as, *ebpB* (endocarditis and biofilm-associated pili), *srtC* (Sortase C), *efaA* (endocarditis specific antigen), *slrA* (transcriptional regulator control initiation of biofilm formation), and *bopD* (biofilm on plastic surface/a putative sugar-binding transcriptional regulator) were also confirmed (48). The presence of antimicrobial peptides (AMPs) was checked by tBLASTn search against the Antimicrobial Peptide Database (APD3) (49) at *e*-value 100, alignment identity >85% and query coverage of 100%. The antimicrobial mechanisms of the AMPs were predicted using antiSMASH v5.0 (50) and BAGEL v4.0 (51) web servers.

### Mobile genetic elements

The presence of MGEs was confirmed using the CRISPRFinder v4.2.20 (52). Prediction and annotation of the prophage sequences was done using the PHAST server^1^ (53) and of insertion sequence elements (ISs) was done using the server ISfinder^2^ (54). The Islandviewer tool was used to detect horizontal transfer of genes.

### Virulence factors and antimicrobial resistance genes

The presence of virulence factors was verified using the VFDB server^3^ (55) and of AMR genes using AMR databases such as CARD v3.1.4 (56), ResFinder v4.1 (57), and CBMAR (58). The presence of bacterial drug efflux pump genes was confirmed using BacEffluxPred (59).

### Genome and proteome comparison of *E. faecium* LR13 with other *E. faecium* probiotic strains

The genome of *E. faecium* LR13 was compared with the genomes of other reported probiotics such as *E. faecium* T110, *E. faecium* 170M39, *E. faecium* SP15 *E. faecium*, and a cholesterol-lowering probiotic strain *E. faecium* WEFA23 which assimilated 85% cholesterol *in vitro*; (17) by *in silico* DNA–DNA Hybridization (DDH) using the web server Genome-to-Genome Distance Calculator (version 2.1)^4^ (60). The average nucleotide identity (ANI) was calculated using the ANI Calculator at EZ-Biocloud server (61). The proteomes were compared by clustering and annotating protein sequences using OrthoFinder tool (62) and webMGA server (63), respectively. The clustered ortholog proteins were further divided into three categories using in-house Perl script as: (i) core (protein sequences present in all the five probiotic strains), (ii) accessory (protein sequences present in different strains but not present in all the five strains), and (iii) unique (protein sequences exclusively present in a particular strain). Functional annotation was performed using NCBI’s COG (cluster of orthologous genes) database to calculate abundance of genes pertaining to a COG family/pathway present in a particular cluster using WebMGA server in (i) core, (ii) accessory, and (iii) unique protein cluster. The statistical analysis was performed using WebMGA server to calculate:


Abundance=NumberofgenesthatbelongtoaCOGfamily/

pathway⁢in⁢a⁢cluster

Total number of genes in COG family/pathway in database.

### Genomic comparison of *E. faecium* LR13 with the genomes of pathogenic, non-pathogenic non-probiotic, and probiotic strains

The genome of *E. faecium* LR13 was compared with the genomes of five pathogenic strains: *E. faecium* Aus0085, *E. faecium* 6E6, *E. faecium* DO, *E. faecium* Aus0004, *E. faecium* ATCC70021, and *E. faecium* E39; two non-pathogenic non-probiotic strains: *E. faecium* 64/3 and *E. faecium* ATCC8459 and four probiotic strains: *E. faecium* T110, *E. faecium* 170M39, *E. faecium* SP15, and *E. faecium* WEFA23. The genome sequences of these strains were retrieved from NCBI and were compared using the genome comparator tool available at PubMLST (64) which identifies the core-genome based multiple locus typing (cgMLST) pattern and builds a Neighbor-Net plot.

Also, the genome of *E. faecium* LR13 was compared with the genomes of 317 *E. faecium* strains reported in an earlier study (65) using Average Nucleotide Index (ANI) (66). These 317 strains represented foodborne, gut commensal, pathogenic, non-pathogenic non-probiotic, and probiotic *E. faecium* strains (65). The ANI-based comparison was performed using the FASTANI program (67) that creates a distance matrix of ANI values, followed by divergence calculation (100-ANI data). The divergence values were used to build a dendrogram with the MEGAX software (68) using the neighbor joining (NJ) algorithm (69).

## Results

### Genomic features of *E. faecium* LR13

**FIGURE 1:**
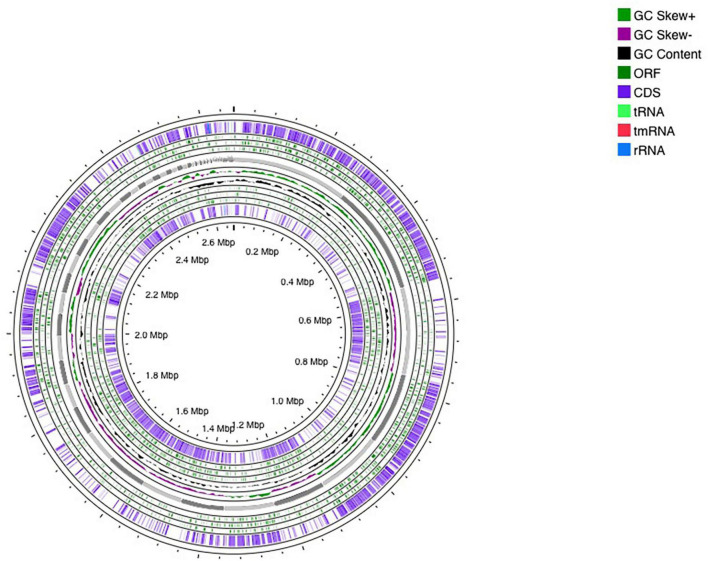
Circular representation of *Enterococcus faecium* LR13 draft genome. The circles were generated using a CG view server.

The genome sequencing of *E. faecium* LR13 was conducted with a sequencing depth of ∼400× which was sufficient for *de novo* assembly. Quality assessment of the reads revealed that Illumina sequencing data was optimal for assembly (Supplementary Links 1, 2) since ∼95% of the reads aligned with the *E. faecium* genome (Table S1 in Supplementary File 1). After trimming, the Phred value of ∼98% reads was >33 indicating a high quality (Table S2 in Supplementary File 1). The overlapping reads were further merged for a better assembly (Table S3 in Supplementary File 1). The final draft assembly showed 97 contigs of varying lengths. Nodes with length 200 bp were discarded and 97 contigs with a length >200 bp were selected for further analysis. Finally, the assembled genome showed 97 contigs, 37.79% GC content and a genome size of 2.66 Mbp. The N50 value of 123,181 bp (Table 1 and Table S4 in Supplementary File 1). Thus, the assembled genome showed 99.63% completeness after marker gene analysis (Table S5 in Supplementary File 1). Within the genome, 2,522 protein-coding genes were identified, of which putative functions were annotated for 1,558 genes while 964 genes were annotated as hypothetical. Also, 18 pseudogenes, 1 tmRNA, 3 rRNAs, and 56 tRNAs were annotated (Table 1 and Figure 1). Genes encoding for proteins residing inside the cellular machinery were analyzed through the RAST subsystem which revealed that 74% of the genes coded for metabolic proteins, protein processing, stress response, DNA and RNA processing and membrane transport, while 26% of the genes were poorly characterized (Fig. S1 in Supplementary Figure 1). The KEGG Automatic Annotation Server (KAAS) revealed enrichment of proteins involved in carbohydrate metabolic pathways in *E. faecium* LR13.

**TABLE 1:** Genomic features of *E. faecium* LR13.

	Features	*E. faecium* LR13 draft genome
Assembly	Genome length	2,665,715 bp (2.66 Mbp)
	GC content (%)	37.79
	N50	123,181
	Largest contig	282,801 bp
Annotations	Number of proteins	2,522
	Number of pseudogenes	18
	Number of rRNAs	3
	Number of tRNAs	53
Genomic signatures	CRISPR array	2
	Number of prophages	10
	IS elements	14
Reference	GenBank accession no.	JANRHE000000000

### Genes for essential probiotic attributes

Several genes responsible for essential probiotic attributes were discerned in the genome of *E. faecium* LR13 genes for acid tolerance: *gadC*, *dltA* and *dltB*, *copA, Dps, clpC, clpE, msrB*, and *terC*; for adhesion and colonization of the gastro-intestinal (GI) tract: *ebpB, srtC, efaA, slrA*, and *bopD*; for hydrolysis of bile salts: *bsh*; for anti-phagocytosis and host-evasion: *cpsA, cpsB, rgpG*, and *epsE*. The complete list of essential probiotic genes discerned in LR13 is presented in Table 2.

**TABLE 2:** Essential and desirable probiotic genes discerned in *E. faecium* LR13.

Probiotic characteristics		Associated genes/proteins	Function	Source/references
Survival in gut		*bsh*	Hydrolysis of conjugated bile salts	(14)
		*dltA*	Gastric acid tolerance	
		*dltB*	Gastric acid tolerance	
		*copA*	Copper-importing P-type ATPase A	
		*gadC*	Protection against extreme acidity of stomach	
		*Dps*	DNA protection during starvation and other stresses	
		*clpC*	Role in persistence of cell Clp ATPase (chaperone)	
		*clpE*	Adaptation to bile Clp ATPase (chaperone)	
		*msrB*	Role in detoxification and persistence	
		*treC*	Osmoprotection	
Antimicrobial property		Enterocin A	Inhibits growth of Gram (+) bacteria	(49, 50)
		Enterocin B	Inhibits growth of Gram (+) bacteria	
		Enterolysin A	Inhibits growth of Gram (+) bacteria	
		Fusaricidin A	Inhibits growth of Gram (+) bacteria and fungi	
		Dosotamide/wollamide	Inhibits growth of Gram (+) bacteria and intracellular pathogens	
		Duracin	Inhibits growth of Gram (+) bacteria by causing cytolysis	
		Champacyclin	Inhibits growth of Gram (+) bacteria	
		Patellamide	Inhibits growth of Gram (+) bacteria	
Essential amino acid biosynthesis	Lysine	*Asd*	Aspartate semialdehyde dehydrogenase	(45, 46)
		*yclM*	Aspartokinase	
		*lysA*	Diaminopimelate decarboxylase	
		*dapF*	Diaminopimelate epimerase	
		*dapB*	Dihydrodipicolinate reductase	
		*dapA*	Dihydrodipicolinate synthetase	
		*dapE*	Succinyl-diaminopimelate desuccinylase	
	Aromatic acid precursor (chorismate) biosynthesis	*aroA*	3-phosphoshikimate 1-carboxyvinyltransferase	
		*aroB*	3-dehydroquinate synthase	
		*aroD*	3-dehydroquinate dehydratase	
		*aroE*	Shikimate biosynthesis protein AroDE	
		*aroF*	3-deoxy-7-phosphoheptulonate synthase	
		*aroC*	Chorismate synthase	
		*aroE*	Shikimate dehydrogenase	
		*aroK*	Shikimate kinase	
	Phenylalanine and tyrosine	*hisC*	Histidinol-phosphate aminotransferase	
		*pheA*	Prephenate dehydratase	
Biofilm formation		*ebpB*	Endocarditis and biofilm-associated pili (adhesion)	(48, 55)
		*srtC*	Sortase C/an enzyme that anchors surface proteins to the cell wall (adhesion)	
		*efaA*	Endocarditis specific antigen (adhesion)	
		*slrA*	Role in adhesion	
		*bopD*	Biofilm on plastic surface/a putative sugar-binding transcriptional regulator (biofilm formation)	
Antiphagocytic activity		*cpsA/uppS*	Host immune evasion	(55)
		*cpsB/cdsA*	Host immune evasion	
Iron uptake		*vctC*	Iron acquisition	(55)
Immune evasion		*cps2T*	Capsule (host immune evasion)	(55)
		*rgpG*	Capsule (host immune evasion)	
		*epsE*	Polysaccharide capsule (host immune evasion)	
Peptidases/proteases		*htrA/degP*	Serine proteases	(47)
		*pepA, pepF, pepT, pepV, pepO, pepQ, pepS, pepF1*	Role in bacterial growth	
Sugar metabolism (PTS)		*ptsG_1, ptsG_2, ptsG_3, ptsH, ptsI, ptsP*	Adaptation to intestinal niche	(47)

### Genes for additional metabolic benefits

Genes that confer additional metabolic benefits on the host, genes for essential amino acid biosynthesis, iron acquisition, carbohydrate metabolism and transport were discerned in *E. faecium* LR13 have been discussed below:

#### Genes for biosynthesis of essential amino acids

**FIGURE 2:**
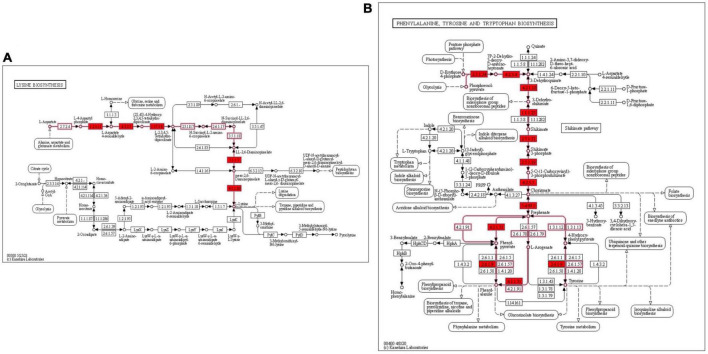
KEGG metabolic pathway for biosynthesis of essential amino acids lysine and aromatic amino acids in *E. faecium* LR13 **(A)** lysine biosynthesis genes are highlighted in red **(B)** phenylalanine and tyrosine biosynthesis genes are highlighted in red. The pathway maps were created using the KEGG database.

Genes encoding the enzymes involved in biosynthesis of host essential amino acids such as lysine, phenylalanine and tyrosine were discerned in LR13. Genes encoding for enzymes involved in biosynthesis of lysine such as *asd* (encodes for aspartate semialdehyde dehydrogenase), *yclM* (encodes for aspartokinase), *lysA* (encodes for diaminopimelate decarboxylase), *dapF* (encodes for diaminopimelate epimerase), *dapB* (encodes for dihydrodipicolinate reductase, *dapA* (encodes for dihydrodipicolinate synthase), and *dapE* (encodes for succinyl-diaminopimelate desuccinylase) were discerned. Also, precursors for biosynthesis of aromatic amino acids (phenylalanine and tyrosine) *aroA* (encodes for 3-phosphoshikimate 1-carboxyvinyltransferase), *aroB* (encodes for 3-dehydroquinate synthase), *aroC* (encodes for chorismate synthase), *aroD* (encodes for 3-dehydroquinate dehydratase), *aroE* (encodes for shikimate dehydrogenase), *aroF* (encodes for 3-deoxy-7-phosphoheptulonate synthase), *aroK* (for shikimate kinase), *hisC* (encodes for histidinol-phosphate aminotransferase, and *pheA* (prephenate dehydratase) were discerned. The complete biosynthetic pathways for lysine and aromatic amino acids depicting the role of these genes are shown in Figures 2A, B.

#### Carbohydrate metabolism and transport genes

KEGG Automatic Annotation Server annotations revealed an abundance of genes related to carbohydrate metabolism in LR13. Also, a variety of genes related to sugar metabolism such as di-, mono-, and oligosaccharides were observed. RAST, PROKKA, and PGAP annotations revealed genes related to glycolysis, pentose phosphate, pyruvate metabolism pathways, etc. Also, genes encoding for carbohydrate transport systems such as phosphoenolpyruvate-dependent sugar phosphotransferase (sugar PTS) genes: *ptsG_1, ptsG_2, ptsG_3*, gene for phosphocarrier protein (*ptsH*), phosphoenolpyruvate-protein phosphotransferase genes *ptsI* and *ptsP* were discerned in LR13 (Table 2).

#### Genes for iron acquisition and proteolytic systems

The gene *vctC* which encodes for the iron chelate ABC transporter ATP-binding protein was discerned in LR13. Also, genes for serine proteases and metallopeptidases such *pepA, pepF, pepT, pepV, pepO, pepQ, pepS*, and *pepF1* were discerned in LR13 (Table 2).

### Genes associated with stress response

Genes encoding stress response such as heat shock proteins of the chaperone class III Clp proteases ClpC and E were discerned in LR13. Also, *dps* which encodes a DNA-binding protein that helps in DNA protection and in universal stress; *msrB* which encodes a protein that plays role in detoxification and *treC* which encodes for proteins mainly involved in osmoprotection were discerned (Table 2).

### Genes encoding for bacteriocins and antimicrobial peptides

**FIGURE 3:**
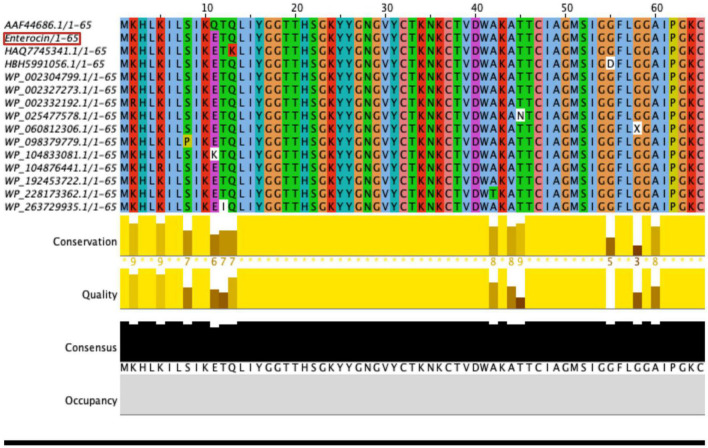
Multiple sequence analysis (MSA) of *E. faecium* LR13 enterocin A (red) with other enterocin A using Muscle tool (111).

*Enterococcus faecium* LR13 harbored genes encoding for three bacteriocins and five antimicrobial peptides (AMPs). The three bacteriocin genes exhibited 100% identity with the reported enterocin A (Figure 3), 97% identity with the enterocin B (Supplementary Figure 5), and 42% identity with enterolysin A (Supplementary Figure 4) gene sequences, respectively. The genes encoding for the five AMPs, fusaricidin A, dosotamide/wollamide, duracin, champacyclin, and patellamide exhibited 100% query coverage with Antimicrobial Peptide Database (APD3) records. The identity with APD3 records was 96.42% for fusaricidin A and dosotamide/wollamide, 87.50% for champacyclin and patellamide and 96.42% for duracin (Table 2).

### Mobile genetic elements

Several MGEs such as plasmids, bacteriophages, and transposons were found in *E. faecium*. The identification of the mobilome in the chromosomal genome was based on screening for CRISPR-Cas elements. No CRISPR and *cas* gene clusters were identified. A total of four prophage regions were detected, of which two of 42.8 and 33.4 kb were intact (PHASTER score ≥100), while two of 20.4 and 15.7 kb were incomplete (PHASTER score ≤50), comprising 4% of the chromosomal genome (Fig. S2 in Supplementary Figure 1). In total, 14 Insertion sequences (ISs) were detected (ISCac2, ISSsu9, ISAac3, ISEfa4, IS21, ISEf1, ISEfa13, IS1485, ISEfa10, ISLca1, IS30, IS6, ISAhy2, and ISBs2) with no associated virulence or AMR gene.

**FIGURE 4:**
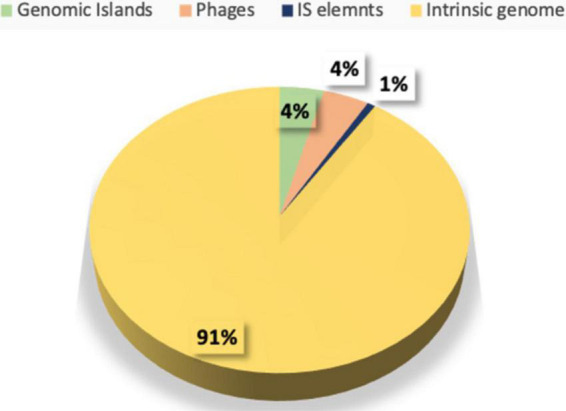
Distribution of various mobile genetic elements like insertion sequences (ISs), genomic islands (GIs), phage (Φ) elements in the genome of *E. faecium* LR13.

When compared with a commercially available probiotic strain *E. faecium* T110, three Genomic islands (GIs) were identified in the genome of LR13, representing 4% of the aligned genome (the chromosomal region). Annotation of GIs revealed that GI1 was composed mainly of ribosomal proteins and transporter genes, GI2 of ISEfa13 element and GI3 of hypothetical proteins (Supplementary Table 2). None of the probiotic, bile salt hydrolase, virulence or AMR genes were found on the GIs of LR13. The MGEs discerned in LR13 are depicted in Figure 4.

### Virulence factors, AMR, and antibiotic efflux pump genes

None of the genes for virulence factors (VFs), antibiotic resistance or efflux of antibiotics were discerned in LR13. However, two AMR genes were found, *msrC* which confers resistance for macrolide*s* and, *aac (6′)-Ii* which confers resistance for fluoroquinolones and aminoglycosides. ResFinder revealed that none of the AMR gene was an acquired resistance gene or present on the plasmids.

### Comparative genomics and proteomics of *E. faecium* LR13 with other *E. faecium* probiotic strains

#### Genome comparison

The genome comparison of LR13 with other probiotic strains revealed its genomic proximity with the general probiotic strains in the order: DDH = 60.10% and ANI = 94.85% with strain T110, DDH = 59.90% and ANI = 98.91% with strain SP15, DDH = 56.60% and ANI = 94.95% with strain 170M39 (Fig. S3 in Supplementary Figure 1). However, LR13 exhibited high genomic relatedness with the cholesterol lowering strain WEFA23 with DDH = 91.70% and ANI = 98.91% (Fig. S3 in Supplementary Figure 1).

#### Proteome comparison

Comparison of the proteome of LR13 with the proteomes of the strains WEFA23, T110, 170M39, and SP15 revealed a total of 3,741 Cluster of Orthologous Genes (COG), of which 1,807 (48.3%) clusters belonged to the core proteome, 863 (23%) to the accessory, and 1,070 (28.6%) to the unique proteome. Overall representation of the COG clusters present in the core, accessory and unique proteome is shown in Fig. S4 in Supplementary Figure 1. Further analysis of the COG clusters revealed that 1,897 proteins were core proteins, 2,475 were accessory proteins, and 1,070 were unique proteins. The strain specific proteins in LR13, WEFA23, T110, 170M39, and SP15 were 716, 48, 83, 131, and 92, respectively. OrthoFinder revealed that 13.7 % of the core, 10.6% of the accessory, and 14.3% of the unique proteins were hypothetical and uncharacterized.

#### Core proteome analysis

OrthoFinder revealed that in the core proteome of all the five probiotic strains, genes corresponding to 1,654 proteins were present as a single copy and 243 proteins as multiple copies. Functional annotation of the core proteins revealed presence of various functional categories within each COG class *viz*, cell growth, DNA replication, recombination and repair, transcription, translation, ribosomal structure and biogenesis, carbohydrate, nucleotide, lipid and amino acid metabolism and transport, defense mechanism, signal transduction, and various transporters. The highest number of core proteins of all the probiotic strains belonged to the COG cluster carbohydrate metabolism and transport (11.4% proteins), followed by translation, ribosomal structure and biogenesis (9% proteins), amino acid transport (7% proteins), nucleotide transport (4% proteins), and coenzyme transport and metabolism (2% proteins). The complete list of core proteins and their corresponding genes is provided in Supplementary Table 3.

#### Accessory proteome analysis

Overall functional annotation of the accessory genes/proteins of all the five probiotic strains revealed two important COG subsystems: (a) carbohydrate metabolism and transport with 30% abundance, (b) DNA transcription with 8% abundance. The complete list of accessory proteins and their corresponding genes is provided in Supplementary Table 4.

#### Unique proteome analysis

In the unique proteome of all the five probiotic strains 18% abundance was observed for DNA replication, recombination and repair proteins, especially the plasmid maintenance proteins, helicases, and transcriptional regulators. This was followed by hypothetical proteins or proteins with unknown functions (14.3% abundance), followed by proteins of the COG category cell wall/membrane/envelope biogenesis ubiquinone, cytochromes, and Phosphotransferase system PTS proteins (12.7% abundance). The unique proteins and their corresponding genes in the probiotic strains are enlisted in Supplementary Table 5. The overall representation of COG in core, accessory and unique proteome is depicted in Supplementary Figure 4.

### Comparative proteome analysis of cholesterol lowering probiotic strains with general probiotic strains

**FIGURE 5:**
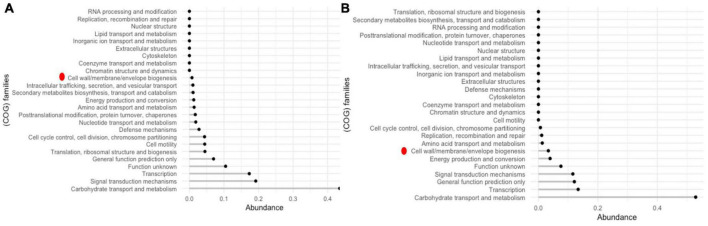
COG families of accessory proteins **(A)** general *E. faecium* probiotic strains – T110, 170M39, and SP15LR13 and **(B)** cholesterol-lowering *E. faecium* probiotic strains LR 13 and WEFA23. Red dot represents the cell wall/membrane/envelope biogenesis COG family proteins which exhibited a fourfold abundance in LR13 and WEFA23.

Comparison of the proteomes of cholesterol lowering (LR13 and WEFA23) and general probiotic strains (T110, 170M39, and SP15) revealed that 52% of the proteins of the hypo-cholesterolemic strains were abundant in the COG category carbohydrate metabolism and transport while 17% of the proteins of the general probiotic strains were abundant in DNA transcription (Figure 5). In hypo-cholesterolemic strains, 3% of the proteins were involved in the COG category cell wall/membrane/envelope biogenesis, while in the general probiotic strains only 0.7% of the proteins were involved in this COG category. Twenty-one proteins were found to be exclusively present in hypo-cholesterolemic strains (LR13 and WEAF3) which were absent in general probiotic strains (T110, SP15, and 17OM9). The details of these proteins are presented in Table 3.

**TABLE 3:** Details of the proteins discerned through COG-clustering as exclusive to hypo-cholesterolemic probiotics *E. faecium* LR13 and WEAF3.

S.No.	UniProtKB IDs	Refseq protein ID/gene ID	Protein name	Gene Ontology (biological process)	Gene Ontology (cellular component)	Gene Ontology (molecular function)	NCBI CDD
1	A0A828ZLC3	WP_002285944.1	Antibiotic biosynthesis monooxygenase	NA	NA	Monooxygenase activity [GO:0004497]	Antibiotic biosynthesis monooxygenase family protein
2	Q3Y302	WP_002286153.1 (Uup)	ABC transporter ATP-binding protein	Transmembrane transport [GO:0055085]	NA	ATP binding [GO:0005524]; transmembrane transporter activity [GO:0022857]	Transmembrane transporter activity
3	Q9KRT5	WP_002287292.1 (UgpE)	Sugar ABC transporter permease. Inner membrane component transport system.	Transmembrane transport [GO:0055085]	Integral component of membrane [GO:0016021]; plasma membrane [GO:0005886]	Integral component of membrane [GO:0016021]; plasma membrane [GO:0005886]; transmembrane transport [GO:0055085]	sn-Glycerol-3-phosphate ABC transporter permease UgpE is part of the binding-protein-dependent transport system for sn-glycerol-3-phosphate; probably responsible for the translocation of the substrate across the membrane
4	P79303	WP_002287294.1 (UgpA)	ABC transporter permease	Transmembrane transport [GO:0055085]	Integral component of membrane [GO:0016021]; plasma membrane [GO:0005886]	Integral component of membrane [GO:0016021]; plasma membrane [GO:0005886]; transmembrane transport [GO:0055085]	ABC transporter permease is the transmembrane subunit (TM) found in periplasmic binding protein (PBP)-dependent ATP-binding cassette (ABC)
5	U2Q622	WP_002288809.1	BglG family transcriptional antiterminator	Regulation of transcription, DNA-templated [GO:0006355]	NA	RNA binding [GO:0003723]; regulation of transcription, DNA-templated [GO:0006355]	Transcriptional antiterminator (transcription)
6	Q3XYJ2	WP_002288811.1	Carbohydrate deacetylase (protein YebG)	Polysaccharide catabolic process [GO:0000272]	NA	Hydrolase activity, acting on carbon-nitrogen (but not peptide) bonds, in linear amides [GO:0016811]; metal ion binding [GO:0046872]; polysaccharide catabolic process [GO:0000272]	Carbohydrate deacetylase catalyzes the deacetylation of acetylated carbohydrates as Chitooligosaccharides at the nonreducing N-acetylglucosamine residue, an important step in the degradation of oligosaccharides
7	Q3XYI6	WP_002288819.1	Glycosyl transferase, MGT family	NA	NA	Glycosyltransferase activity [GO:0016757]	UDP-glucuronosyl and UDP-glucosyl transferase, located on cell surfaces
8	U2NW17	WP_002289597.1	LuxR family DNA-binding response regulator	Phosphorelay signal transduction system [GO:0000160]; 9 regulation of transcription, DNA-templated [GO:0006355]	NA	DNA binding [GO:0003677]	DNA-binding effector domain of two-component system response regulators
9	D5HKK5	WP_002299677.1 (BaeS)	Histidine kinase (EC 2.7.13.3)	NA	Integral component of membrane [GO:0016021]	Integral component of membrane [GO:0016021]; phosphorelay sensor kinase activity [GO:0000155]	Integral component of membrane; phosphorelay sensor kinase activity.
10	A0A133CIV3	WP_002301171.1	MBL fold metallo-hydrolase (metal-dependent hydrolase)	NA	NA	DNA binding [GO:0003677]	
11	P96622	WP_002302307.1 (MazF)	MazF family toxin-antitoxin system, toxin component	mRNA catabolic process [GO:0006402]	NA	DNA binding [GO:0003677]	PemK-like, MazF-like toxin of type II toxin-antitoxin system
12	P9WQD5	WP_025477609.1 (TesA)	SGNH/GDSL hydrolase family protein	Lipid biosynthetic process [GO:0008610]	Plasma membrane [GO:0005886]	Hydrolase activity [GO:0016787]	Involved in the surfactin biosynthesis pathway
13	Q7UGU0	WP_002307216.1	GNAT family N-acetyltransferase	N-terminal protein amino acid acetylation [GO:0006474]	Ribosomal-protein-alanine N-acetyltransferase complex [GO:0009323]	N-acetyltransferase activity [GO:0008080]	GNAT family N-acetyltransferase catalyzes the transfer of an acetyl group from acetyl-CoA to a substrate
14	Q8P4Q8	WP_002328020.1 (Lrp)	Winged helix-turn-helix transcriptional regulator	Regulation of transcription, DNA-templated [GO:0006355]	Cytosol [GO:0005829]	Sequence-specific DNA binding [GO:0043565]	Leucine-responsive transcriptional regulator Lrp mediates a global response to leucine
15	A0A3N4B2U6	WP_002328308.1 (BglB)	Glycoside hydrolase family 1 protein	Carbohydrate metabolic process [GO:0005975]	NA	Beta-glucosidase activity [GO:0008422]; scopolin beta-glucosidase activity [GO:0102483]	Beta-glucosidase/6-phospho-beta-glucosidase/beta-galactosidase (carbohydrate transport and metabolism)]
16	B5YIF1	WP_010725405.1 (Smc)	Hypothetical protein	DNA replication [GO:0006260]	Chromosome [GO:0005694]; cytoplasm [GO:0005737]	ATP binding [GO:0005524]; ATP hydrolysis activity [GO:0016887]; DNA binding [GO:0003677]	
17	P11989	WP_033603018.1	HTH domain-containing protein	Positive regulation of transcription, DNA-templated [GO:0045893]	NA	RNA binding [GO:0003723]	Transcriptional anti terminator (Transcription)
18	UPI000C77AD8E	WP_101735266.1 (VanY)	D-alanyl-D-alanine carboxypeptidase DdcY	NA	NA	NA	D-alanyl-D-alanine carboxypeptidase, which removes C-terminal D-alanyl residues from sugar-peptide cell wall precursors
19	UPI000C775590	WP_101735336.1	DNA primase family protein	NA	NA	NA	NA
20	UPI0002A38D29	WP_002328046.1	Mannosyl-glycoprotein endo-beta-N-acetylglucosaminidase	NA	NA	NA	Hydrolyze chitin, an abundant polymer of beta-1,4-linked N-acetylglucosamine (GlcNAc) which is a major component of the cell wall of bacteria
21	P9WK41	LprI	Putative lipoprotein	NA	Cell surface [GO:0009986]; extracellular region [GO:0005576]; plasma membrane [GO:0005886]	NA	NA

### Core-genome based multiple locus typing of *E. faecium* LR13 pathogenic, non-pathogenic non-probiotic, and probiotic strains

The cgMLST of the 12 *E. faecium* strains comprising pathogenic, non-pathogenic non-probiotic, and probiotic strains revealed three clusters, (i) pathogenic strains, (ii) non-pathogenic, non-probiotic strains and, (iii) probiotic strains. *E. faecium* LR13 belonged to the probiotic cluster (Supplementary Figure 2 and Table S6 in Supplementary File 1).

### Genomic similarity of LR13 with other *E. faecium* strains

Calculation of the nucleotide-level genomic similarity of LR13 with 317 *E. faecium* strains reported in an earlier study (65) revealed that *E. faecium* LR13 belonged to the cluster containing gut-associated strains with the ANI value above 99.5 (Supplementary Figure 3).

## Discussion

Most of the probiotic *E. faecium* strains are isolated from animal/human gut and fermented foods (70, 71). *E. faecium* LR13 was the first rhizospheric isolate which not only exhibited several probiotic attributes but also bile salt hydrolase activity and hypocholesterolemic activity *in vitro* (18). Thus, a complete understanding of the genomic features of this strain and comparison with other probiotic and pathogenic *E. faecium* strains can help in understanding the genomic relatedness/differences in probiotic/non-probiotic environmental or animal isolates.

The essential attributes possessed by a probiotic strain included the genes that helps in adherence, competence, survival, tolerance, and persistence in harsh conditions of the GI-tract. Genome sequencing and computational analysis revealed that the probiotic attributes exhibited phenotypically by *E. faecium* LR13 co-related exactly with the presence of the respective genes. Genes encoding for essential probiotic attributes such as acid tolerance, (*dltA*, *dltB*, and *gadC*), bile resistance (*clpC*, *clpE*, and *dps*), self-aggregation (*bopD*), adhesion and colonization (*ebpB*, *srtC*, *efaA*, and *slrA*) (48) hydrolysis of conjugated bile salts (*bsh*), were discerned in LR13.

Also, several other genes beneficial in populating the host GI-tract were discerned in LR13 such as genes for colonization and host immune evasion (*cpsA*, *cpsB*, *cps2T*, *rgpG*, and *epsE*), persistence and detoxification (*msrB*) and, growth and osmoprotection (*treC*) (14). Also, several genes encoding serine proteases and metallopeptidases such as *pepA, pepF, pepT, pepV, pepO, pepQ, pepS*, and *pepF1* were discerned in LR13. The peptidases/proteases helps in bacterial growth inside the host (47). A variety of genes related to sugar metabolism, glycolysis, pentose phosphate, and pyruvate metabolism pathways were discerned. An earlier study also reported that genes for carbohydrate metabolism are more abundant in probiotic strains than the pathogenic strains (72). Genes encoding carbohydrate transport systems including phosphoenolpyruvate-dependent sugar phosphotransferases (sugar PTS), PTS system glucose-specific EIICB component (*ptsG_1, ptsG_2*, and *ptsG_3*), phosphocarrier protein (*ptsH*), phosphoenolpyruvate-protein phosphotransferase (*ptsI*) and (*ptsP*) were identified in LR13. The PTS component genes are essential for adaptation in the intestinal niche and their presence is frequently reported in gut/commensal bacteria (47). The presence of these genes suggests that LR13 might fairly survive and transit in the GI-tract without the need for any protective encapsulation.

Besides, genes whose products might confer some additional metabolic benefits on the host were discerned in LR13. These included genes for biosynthesis of amino acids, lysine, phenylalanine, and tyrosine which are essential amino acids. Essential amino acids cannot be synthesized by the human body and, since they are necessary for the normal functioning of the cells they have to be taken with the diet. Besides, these amino acids are precursors for short-chain fatty acids which play diverse physiological roles in human health, production of vitamins, lipids, and energy (73). The presence of iron-chelate ABC transporter ATP-binding protein, *vctC* in the genome of *E. faecium* LR13 might also have metabolic implications for the host because the host iron is used by several pathogenic bacteria for colonization and pathogenesis (74, 75). The presence of iron uptake genes in probiotics can pose competition to the pathogens for the host-iron and thus reduce the pathogen population inside the host. As was also observed in the case of probiotic *Escherichia coli Nissle*, which helped in reducing the host colonization by pathogenic *Salmonella typhimurium* (76).

*Enterococcus faecium* LR13 harbored genes encoding for three bacteriocins and five AMPs. The three bacteriocin genes belonged to the bacteriocin-families, enterocin A, enterocin B, and enterolysin A, respectively. Interestingly, the enterocin A of LR13 was identical with an earlier reported enterocin from *E. faecium* 170M39 which exhibited antimicrobial activity against *Listeria monocytogenes* (15). *L. monocytogenes* is a pathogenic food-borne bacteria associated with a serious infection called listeriosis. This suggests that *E. faecium* LR13 might also protect against listeriosis. Besides, the presence of genes encoding AMPs, Fusaricidin A, Dosotamide/wollamide, Duracin, Champacyclin, and Patellamide can play an important role in the development of a healthy microbiome inside the host because AMPs reportedly inhibit the growth of Gram (+) positive bacteria (77).

*Enterococcus faecium* is composed of both pathogenic and non-pathogenic strains (78, 79) and many strains can acquire virulence genes over the course of time. Thus, identification of virulence genes is pivotal before categorizing an *E. faecium* strain as a probiotic. None of the virulence genes prevalent in enterococci, genes encoding for cytolysins, hemotoxins, aggregation, and chemotaxis were discerned in LR13 indicating that it was safe for human consumption. VRE are notorious nosocomial pathogens, hence, potential probiotic *E. faecium* strains should not harbor vancomycin resistance genes (11). Our analysis revealed that LR13 neither harbored any AMR genes reported for pathogenic enterococci (80) nor vancomycin resistance genes.

The MGEs including plasmids, ISs, transposons, bacteriophages, and GIs play an important role in horizontal gene transfer and transmission of virulence/antibiotic resistance genes (81). Reportedly, the MGEs of probiotic enterococci are different from pathogenic enterococci (72). ISs, ISEfa11, and ISEfa5 have been reportedly associated with vancomycin resistant genes *vanS*, *vanX*, and *vanY* of *E. faecium* (82). Bacteriophages or prophages provide new genetic characteristics to the host and increase virulence of pathogenic bacteria, *Vibrio cholerae* (83), *E. coli* (84), and *Corynebacterium diphtheriae* (85). Genes present on GIs are usually mobile genes representing the acquired traits (26). Thus, it was necessary to ascertain if the antibiotic resistance, virulence and/or probiotic traits of LR13 were intrinsic or acquired traits. Our results revealed that LR13 did not carry plasmid, IS element, transposon, bacteriophage or GI-linked antibiotic resistance or virulence genes. Moreover, none of the genes for probiotic attributes were associated with MGEs suggesting that the probiotic attributes exhibited by LR13 were intrinsic traits and not acquired.

The genomic comparison of LR13 with the genomes of foodborne, gut commensal, pathogenic, non-pathogenic non-probiotic, and probiotic *E. faecium* strains revealed that it lies within the cluster of gut commensal strains. The cgMLST based genotyping further strengthened the fact that LR13 was quite distinct from pathogenic and laboratory strains. These findings are in concordance with an earlier study which also reported that food-grade and nosocomial enterococci strains are genetically distinct (20).

Cholesterol-assimilation capability has been reported for only a few probiotic *Lactobacillus plantarum* and *E. faecium* strains (17, 18, 72, 86, 87). Comparative genomic and proteomic analysis of the cholesterol-lowering probiotic *E. faecium* strains (LR13 and WEFA23) and general *E. faecium* probiotic strains (T110, 170M39, and SP15) revealed that LR13 was genetically more related to the cholesterol-lowering strain WEFA23 (DDH = 91.70% and ANI = 98.91%), than other probiotic strains.

Comparison of the COG categories revealed at least a 4.28-fold abundance of cell wall/membrane/envelope biogenesis COG category proteins in cholesterol lowering probiotics than general probiotic strains. GO-based functional annotation and literature survey of the proteins exclusively discerned in hypo-cholesterolemic probiotic strains (LR13 and WEAF3) revealed that of the 21 proteins, 17 proteins might be directly/indirectly related to cholesterol assimilation. The four proteins for which a correlation with cholesterol-assimilation could not be discerned were, A0A133CIV3, P96622, B5YIF1, and UPI000C775590. A0A133CIV3 was a MBL fold metallo-hydrolase (metal-dependent hydrolase), P96622 – a MazF family toxin-antitoxin system protein, B5YIF1 – a hypothetical protein and UPI000C775590 – a DNA primase family protein. Interestingly of the remaining 17 proteins, 14 proteins might help directly in cholesterol-assimilation by employing either of the three mechanisms, (i) production of short chain fatty acids (SCFAs), (ii) lipid (sterol) transport and membrane stabilization, and (iii) bile salt hydrolase (*bsh*) activity (Graphical Abstract).

### Proteins involved in production of short chain fatty acids

Short chain fatty acids are fatty acids with less than 6 carbon atoms. These are produced in the human gut by the gut microbes as a by-product of breakdown/fermentation of dietary fibers (88). SCFAs are transported from the gut to the liver where they inhibit the HMG-CoA reductase enzyme (3-hydroxy-3-methylglutaryl-CoA) which converts HMG-CoA to mevalonic acid (89). Interestingly, this step is the rate limiting step of the cholesterol biosynthesis pathway and also the target of statins (90). GO annotations, NCBI Conserved Domain Database (CDD) search and literature evidences revealed that six proteins, Q3XYJ2 (yebG), Q3XYI6, P9WQD5 (TesA), UPI0002A38D29 (ENGase), P1198 (BglG), and UPI000C77AD8E (DdcY) were involved in sugar uptake and breakdown/fermentation into SCFAs (Table 3). The protein Q3XYJ2 was a carbohydrate deacetylase, Q3XYI6, a glycosyltransferase, P9WQD5 was a SGNH/GDSL hydrolase family protein, UPI0002A38D29 a mannosyl-glycoprotein endo-beta-N-acetylglucosaminidase, P1198 an RNA binding protein that regulates the expression of genes involved in sugar utilization and UPI000C77AD8E removes D-alanyl residue from sugars. These proteins participate in uptake and breakdown of complex sugars which are fermented to produce SCFAs (91–94).

### Proteins involved in lipid (sterol) transport, membrane stabilization, and binding of cholesterol to the bacterial cell walls

Several studies have indicated that gut bacteria might assimilate cholesterol by binding/attaching to the cell walls and incorporating in their membranes (87, 95). Recently, cholesterol-assimilation was related to the metabolic state of the bacteria and live cells reportedly accumulated more cholesterol than the dead cells (95). GO annotations, NCBI-CDD and literature evidences revealed that Q3Y02, Q9KRT5, P7903, and U2Q622 (membrane transporter/importers) and, U2NW17, D5HKK5, and P9WK41 (membrane stabilization proteins) might be involved in cholesterol uptake and stabilization of bacterial membrane. Q3Y302 was an ABC transporter ATP-binding protein, and Q9KRT5 and P79303 were ABC transporter permeases. ABC transporters are involved in transport of lipids, sterols (96) while Q9KRT5 and P79303 are bacterial importers also known as permeases which transport sugars, ions, and lipids (97). U2Q622 was a BglG family transcriptional anti-terminator which regulates oppA that encodes an oligopeptide transporter (98). U2NW17 was a LuxR family DNA-binding response regulator while D5HKK5 was a histidine kinase. Both U2NW17 and D5HKK5 are putative stress response proteins involved in cell wall homeostasis and regulation of peptidoglycan plasticity (99–101). P9WK41 was a putative lipoprotein that plays an important role in cell wall biosynthesis (102). A recent report has suggested that lipoproteins are important for cholesterol uptake (103).

### Protein involved in bile salt hydrolysis

GO annotations and NCBI-CDD revealed that Q7UGU0 (bshB) was a homolog of the bile salt hydrolase, bshA. Most of the gut microbes usually have one, but some probiotics might harbor multiple BSH homologs (104). Q7UGU0 (bshB) is a GNAT family N-acetyl transferase which deconjugates conjugated bile salts. Since deconjugated bile salts are not as efficiently absorbed in the intestine, they are excreted out in the feces as free bile acids. Thus, deconjugation of bile salts can potentially reduce the serum cholesterol levels by increasing the use of host cholesterol for bile acid synthesis (105). Also, deconjugated bile salts do not efficiently solubilize and absorb lipids resulting in reduced cholesterol solubility and absorption by the intestinal lumen (104).

The other three proteins, A0A828ZLC3, A0A3N4B2U6, and Q8P4Q8 did not exhibit a direct correlation with cholesterol-assimilation but might help in bacteria in other ways. A0A828ZLC3, was an antibiotic biosynthesis monooxygenase protein which initiates degradation of sterol side chain in bacteria (106). A0A3N4B2U6 is a glycoside hydrolase family 1 protein which helps in auto-aggregation of bacterium by linking cell and extracellular molecules of glucan (107). Q8P4Q8 was a winged helix-turn-helix transcriptional regulator which plays an important role in bacterial DNA stabilization (108).

## Conclusion

To summarize, a comprehensive genome analysis of *E. faecium* LR13 revealed that it was genetically related to food grade enterococci and was safe for human consumption. Additionally, besides the usual probiotic benefits it can bestow several other additional metabolic benefits on the host as the genome of LR13 harbored genes for biosynthesis of essential amino acids, carbohydrate metabolism, iron acquisition, and production of bacteriocins and antimicrobial peptides. Cholesterol-assimilation capability has been reported for only a few probiotic *L. plantarum* and two *E. faecium* strains. Among all the probiotic strains tested, LR13 was genetically more related to WEFA23 – another cholesterol assimilating probiotic. Both LR13 and WEFA23 harbored 21 unique proteins which were absent in other probiotic strains. Of these, three proteins were indirectly related to cholesterol-assimilation while 14 proteins might directly help in cholesterol assimilation. However, *in vivo* and mutation-based studies are required to establish the mechanism and identify the precise role of these proteins in cholesterol assimilation. The fact that the general probiotic strains were devoid of proteins directly/indirectly related with cholesterol assimilation, suggests that cholesterol-assimilation might be an inherent, strain specific trait present in a few probiotics with a specific genetic constitution. A recent study identified many cholesterol-interacting microbes suggesting that a specific microbiome can precisely regulate the host-cholesterol homeostasis (109). They also identified a novel mechanism for microbial transformation of dietary cholesterol enabled by the presence of a cholesterol-responsive sulfotransferase in Bacteroides (109). Moreover, the unique proteins identified in this study can serve as biomarkers for discerning/characterizing cholesterol-assimilating probiotics as novel biotherapeutics for treating CVDs. However, more cholesterol assimilating probiotics need to be studied to arrive at some definite conclusion.

## Data availability statement

The datasets presented in this study can be found in online repositories. The names of the repositories and accession number(s) can be found below: National Center for Biotechnology Information (NCBI) GenBank, https://www.ncbi.nlm.nih.gov/genbank/, JANRHE000000000 NCBI Sequence Reads Archive (SRA), https://www.ncbi.nlm.nih.gov/sra, SRR20852288.

## Author contributions

NS designed the experiment. MA performed the bioinformatics analysis. NS and MA analyzed the data and prepared the manuscript. MK conceived the idea. All authors reviewed the manuscript.

## Footnotes

1
http://phast.wishartlab.com/

2
https://isfinder.biotoul.fr/

3
http://www.mgc.ac.cn/VFs/

4
http://ggdc.dsmz.de/

## Supplementary material

Supplementary Figure 1 Phages present in LR13 genome. Figure S2: RAST subsystem analysis. Figure S3: DDH and ANI calculation of other strains represented with LR13 genome. Figure S4: Overall COG representation of core, accessory and unique present in LR13, WEFA23, T110, 170M39, and SP15 probiotic strains.

Supplementary Figure 2 An overview of genome comparison of *E. faecium* LR13 with 317 published genomes of foodborne, gut commensal, pathogenic, non-pathogenic non-probiotic and probiotic *E. faecium* strains. The comparison was performed with an ANI-based approach using FASTANI. *E. faecium* LR13 was found to be related to the gut-associated strains (marked in black color).

Supplementary Figure 3 A neighbor-net view of the core-genome comparative analysis of *E. faecium* LR13 with five pathogenic *E. faecium* strains (Aus0085, 6E6, DO, Aus0004, ATCC70021 and E39), two non-pathogenic non-probiotic strains (64/3, ATCC8459) and four probiotic strains (T110, 170M39, SP15 and WEFA23). The comparison was performed using a genome comparator tool available at PubMLST to discern the cgMLST. *E. faecium* LR13 showed more proximity with the probiotic strains (green color) than non-probiotic non-pathogenic (yellow color) and pathogenic strains (orange color).

Supplementary Figure 4 Multiple sequence analysis (MSA) of *E. faecium* LR13 enterolysin A (Red) with other enterolysin A using Muscle tool.

Supplementary Figure 5 Multiple sequence analysis (MSA) of *E. faecium* LR13 enterolysin B (Red) with other enterolysin B using Muscle tool.

Supplementary File 1 Table S1: BLAST analysis of pair-end reads of *E. faecium* LR13. Table S2: After trimmomatic analysis the reads left for further analysis. Table S3: Reads after merge of overlapping reads. Table S4: N50, GC%, and NG50 value when aligned with the reference *E. faecium* genome. Table S5: Completeness of assembly. Table S6: cgMLST output.

Supplementary File 2 Detailed information of the core genes in the studied genomes.

Supplementary File 3 Detailed information of the accessory genes in the studied genomes.

Supplementary File 4 Detailed information of the unique genes in the studied genomes.

Supplementary File 5 Detailed information of the Genomic Islands in *E. faecium* LR13.

Supplementary Link 1 FastQC output html file (LR13_HHH23DSXX_L3_1_fastqc.html).

Supplementary Link 2 FastQC output html file (LR13_HHH23DSXX_L3_2_fastqc.html).
