# Supplementary material for: Comprehensive genomic analysis of hypocholesterolemic probiotic Enterococcus faecium LR13 reveals unique proteins involved in cholesterol-assimilation
Source: Front Nutr. 2023 Apr 4;10:1082566. doi: 10.3389/fnut.2023.1082566 (PMC10110904; doi:10.3389/fnut.2023.1082566)

Supplementary Fig 1: Overview of subsystem-based analysis to annotate *Enterococcus faecium* LR13 genome. The whole-genome sequence of the strain LR13 was annotated using PATRIC RASTtk server.

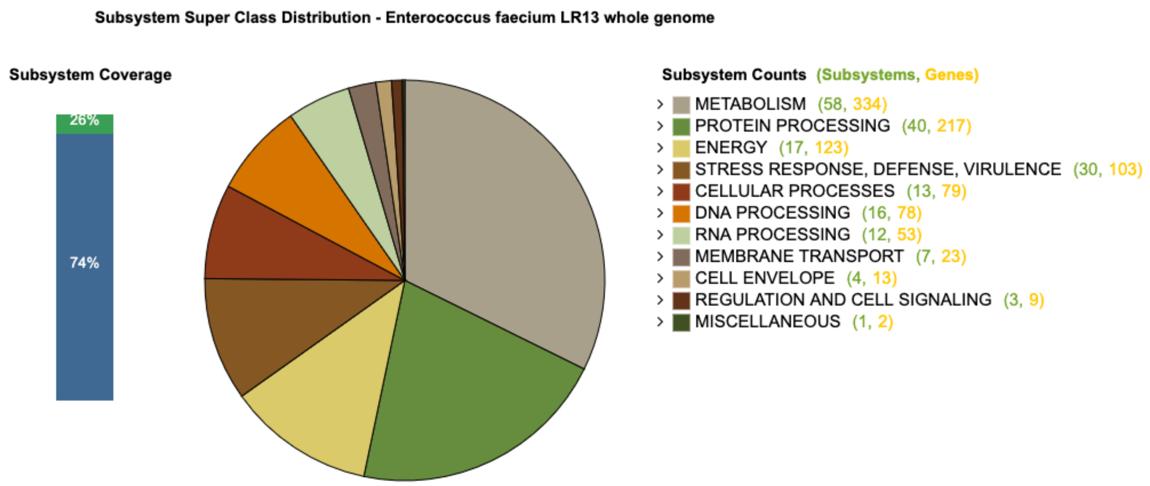

Supplementary Fig 2: 10 prophage regions have been predicted in complete genome using PHAST server, of which 3 regions were intact (Red), 6 regions were incomplete (grey) and 1 regions were questionable (green).

Region	Region Length	Completeness	Score	# Total Proteins	Region Position	Most Common Phage	GC %	Details
NODE_14_length_67016_cov_7.921451								
1	66Kb	intact	150	75	155-66211	PHAGE_Aeromo_AS_gz_NC_042019(71)	41.00%	Show
NODE_18_length_51843_cov_570.805181								
2	42.8Kb	intact	100	63	8203-51076	PHAGE_Lister_B025_NC_009812(5)	34.36%	Show
NODE_19_length_51673_cov_380.136561								
3	33.4Kb	intact	120	51	10766-44203	PHAGE_Bacill_BCJA1c_NC_006557(10)	36.76%	Show
NODE_20_length_49166_cov_8.829045								
4	49Kb	incomplete	60	76	145-49166	PHAGE_Aeromo_AS_gz_NC_042019(63)	41.80%	Show
NODE_28_length_28821_cov_8.721229								
5	28.1Kb	incomplete	50	62	3-28141	PHAGE_Aeromo_Aes012_NC_020879(43)	41.23%	Show
NODE_29_length_21725_cov_3.283444								
6	20.4Kb	incomplete	50	26	2-20415	PHAGE_Salmon_38_NC_029042(10)	45.49%	Show
NODE_34_length_16032_cov_9.050517								
7	15.8Kb	questionable	80	29	1-15845	PHAGE_Aeromo_Aes012_NC_020879(23)	41.53%	Show
NODE_35_length_15953_cov_2.945641								
8	15.7Kb	incomplete	60	26	1-15707	PHAGE_Salmon_38_NC_029042(10)	45.64%	Show
NODE_39_length_12292_cov_2.912321								
9	11.2Kb	incomplete	60	17	1048-12290	PHAGE_Escher_EP75_NC_049433(13)	44.71%	Show
NODE_48_length_8117_cov_3.381716								
10	8.1Kb	incomplete	30	19	8-8115	PHAGE_Salmon_heyday_NC_049500(7)	43.60%	Show

Supplementary Fig. 3: Pan-genomic comparison of *E. faecium* LR13 with other probiotic strains using DNA-DNA Hybridization (DDH) and average nucleotide identity (ANI)

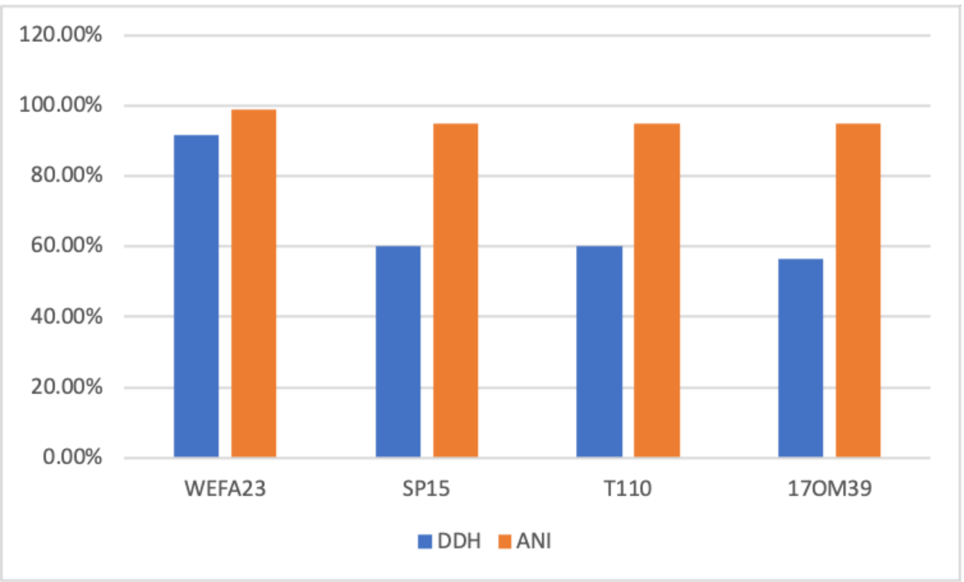

Supplementary Fig 4: Overall COG representation of core, accessory and unique present in LR13, WEFA23, T110, 170M39 and SP15 probiotic strains

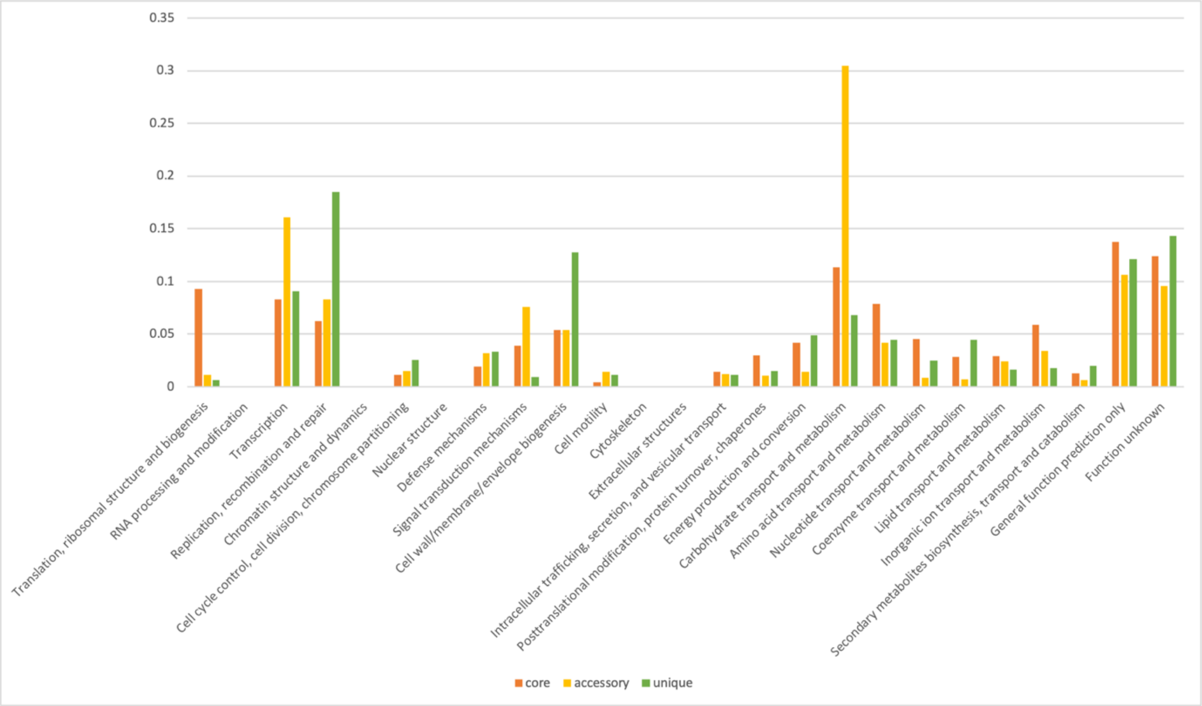

Supplement: Supplementary Figure 1 — Phages present in LR13 genome. Figure S2: RAST subsystem analysis. Figure S3: DDH and ANI calculation of other strains represented with LR13 genome. Figure S4: Overall COG representation of core, accessory and unique present in LR13, WEFA23, T110, 170M39, and SP15 probiotic strains. [file Image_1.pdf]
