# Supplementary figures and images for: Comprehensive genomic analysis of hypocholesterolemic probiotic Enterococcus faecium LR13 reveals unique proteins involved in cholesterol-assimilation
Source: Front Nutr. 2023 Apr 4;10:1082566. doi: 10.3389/fnut.2023.1082566 (PMC10110904; doi:10.3389/fnut.2023.1082566)

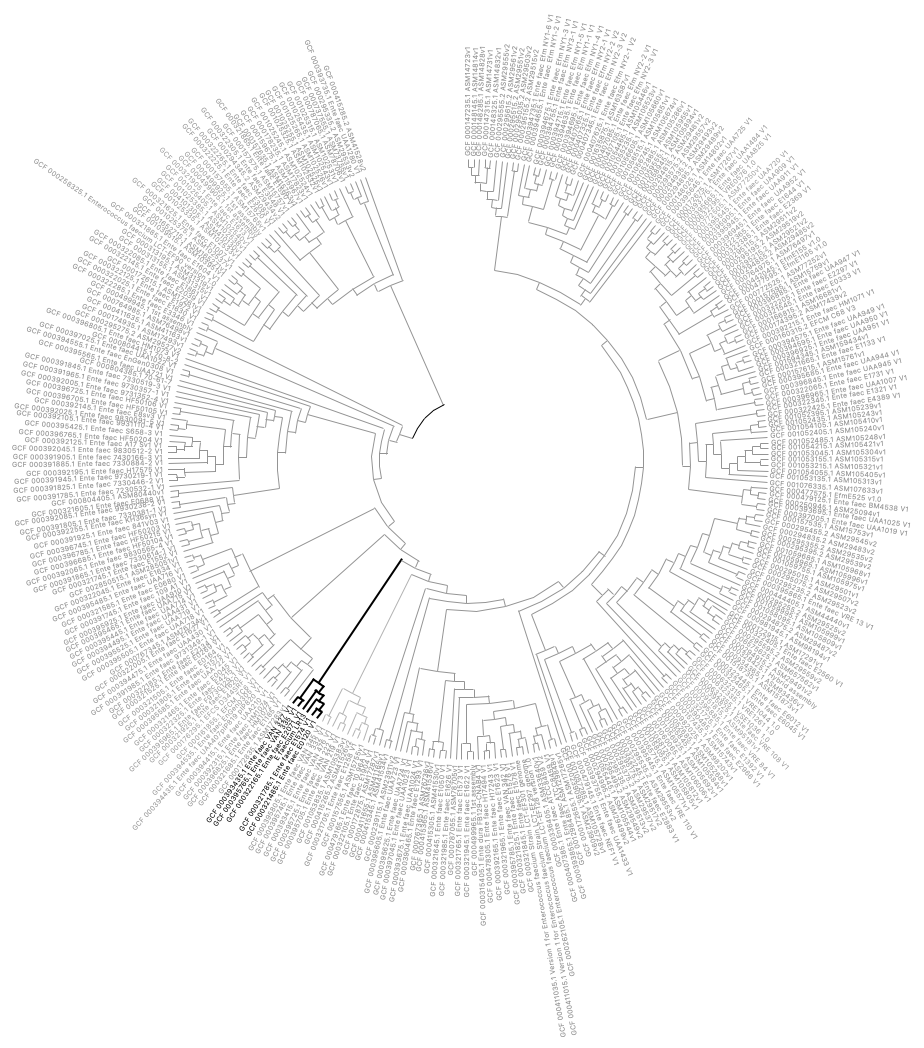

Supplement: Supplementary Figure 2 — An overview of genome comparison of E. faecium LR13 with 317 published genomes of foodborne, gut commensal, pathogenic, non-pathogenic non-probiotic and probiotic E. faecium strains. The comparison was performed with an ANI-based approach using FASTANI. E. faecium LR13 was found to be related to the gut-associated strains (marked in black color). [file Image_2.png]

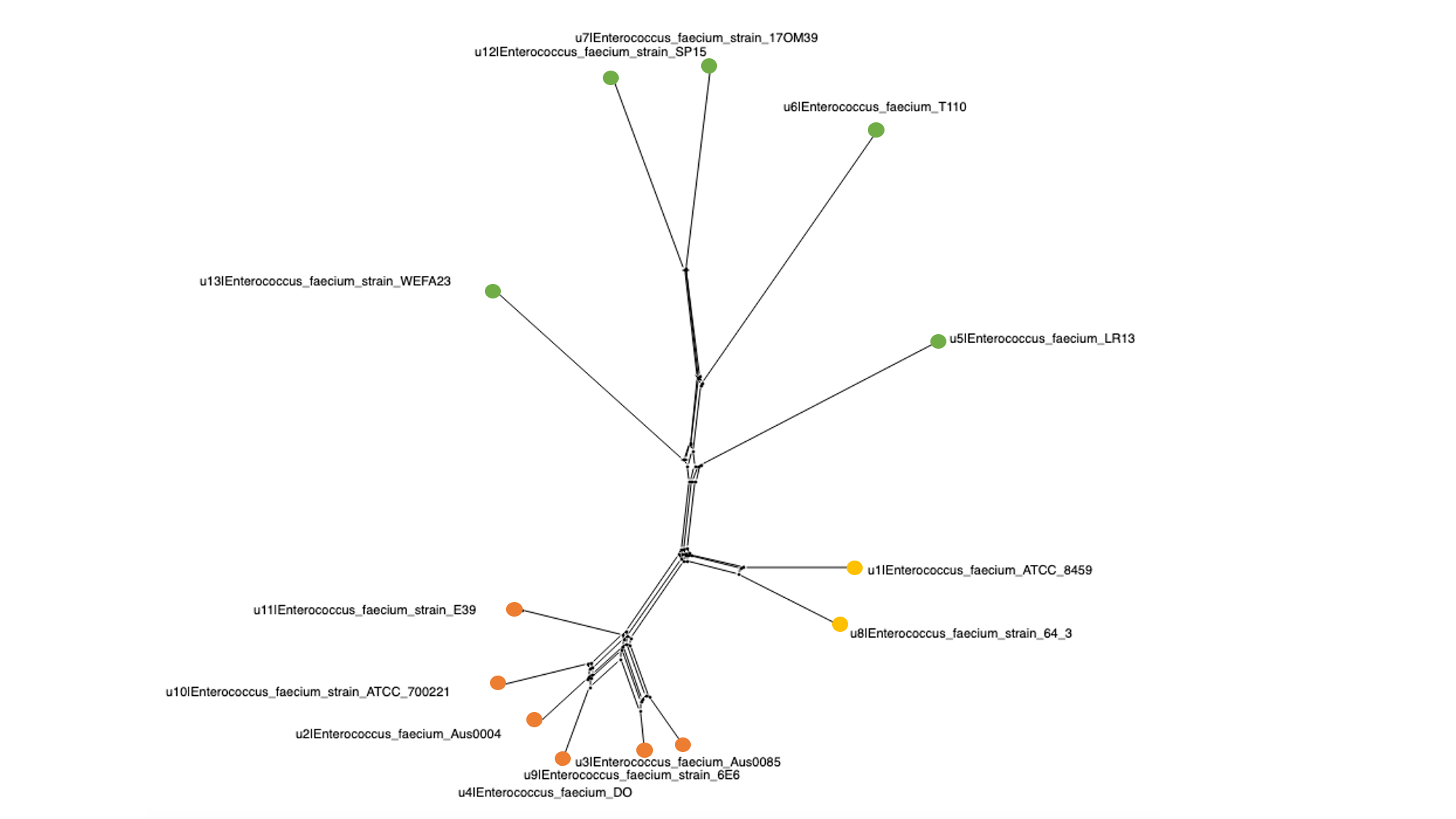

Supplement: Supplementary Figure 3 — A neighbor-net view of the core-genome comparative analysis of E. faecium LR13 with five pathogenic E. faecium strains (Aus0085, 6E6, DO, Aus0004, ATCC70021 and E39), two non-pathogenic non-probiotic strains (64/3, ATCC8459) and four probiotic strains (T110, 170M39, SP15 and WEFA23). The comparison was performed using a genome comparator tool available at PubMLST to discern the cgMLST. E. faecium LR13 showed more proximity with the probiotic strains (green color) than non-probiotic non-pathogenic (yellow color) and pathogenic strains (orange color). [file Image_3.png]

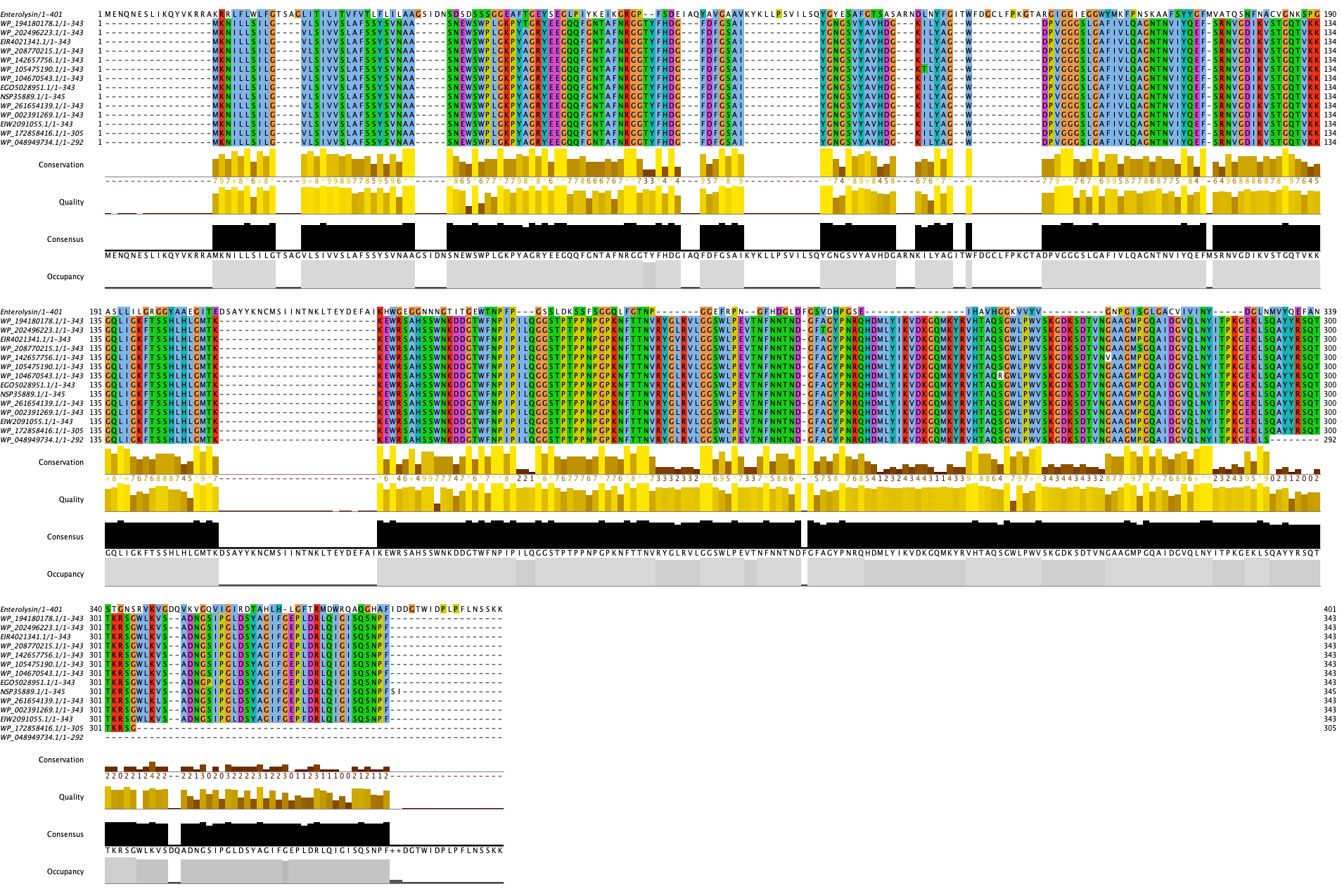

Supplement: Supplementary Figure 4 — Multiple sequence analysis (MSA) of E. faecium LR13 enterolysin A (Red) with other enterolysin A using Muscle tool. [file Image_4.png]

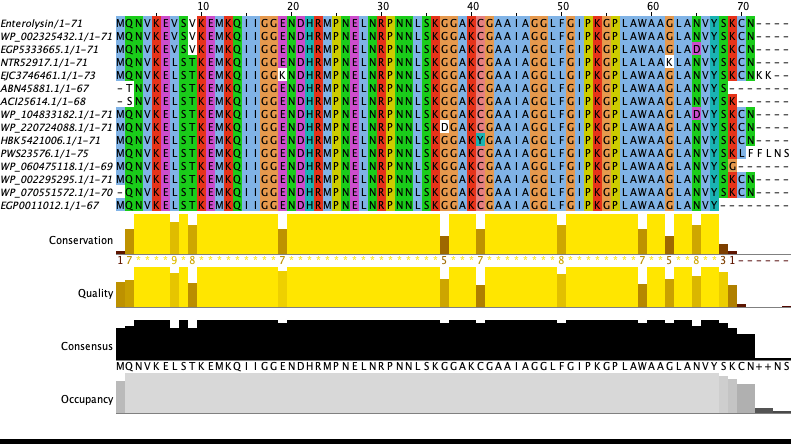

Supplement: Supplementary Figure 5 — Multiple sequence analysis (MSA) of E. faecium LR13 enterolysin B (Red) with other enterolysin B using Muscle tool. [file Image_5.png]
