# Supplementary material for: Comprehensive genomic analysis of hypocholesterolemic probiotic Enterococcus faecium LR13 reveals unique proteins involved in cholesterol-assimilation
Source: Front Nutr. 2023 Apr 4;10:1082566. doi: 10.3389/fnut.2023.1082566 (PMC10110904; doi:10.3389/fnut.2023.1082566)

Summary

- Basic Statistics
- Per base sequence quality
- Per tile sequence quality
- Per sequence quality scores
- Per base sequence content
- Per sequence GC content
- Per base N content
- Sequence Length Distribution
- Sequence Duplication Levels
- Overrepresented sequences
- Adapter Content

Basic Statistics

Measure	Value
Filename	LR13_HHH23DSXX_L3_2.fq
File type	Conventional base calls
Encoding	Sanger / Illumina 1.9
Total Sequences	8994262
Sequences flagged as poor quality	0
Sequence length	150
%GC	38

Per base sequence quality

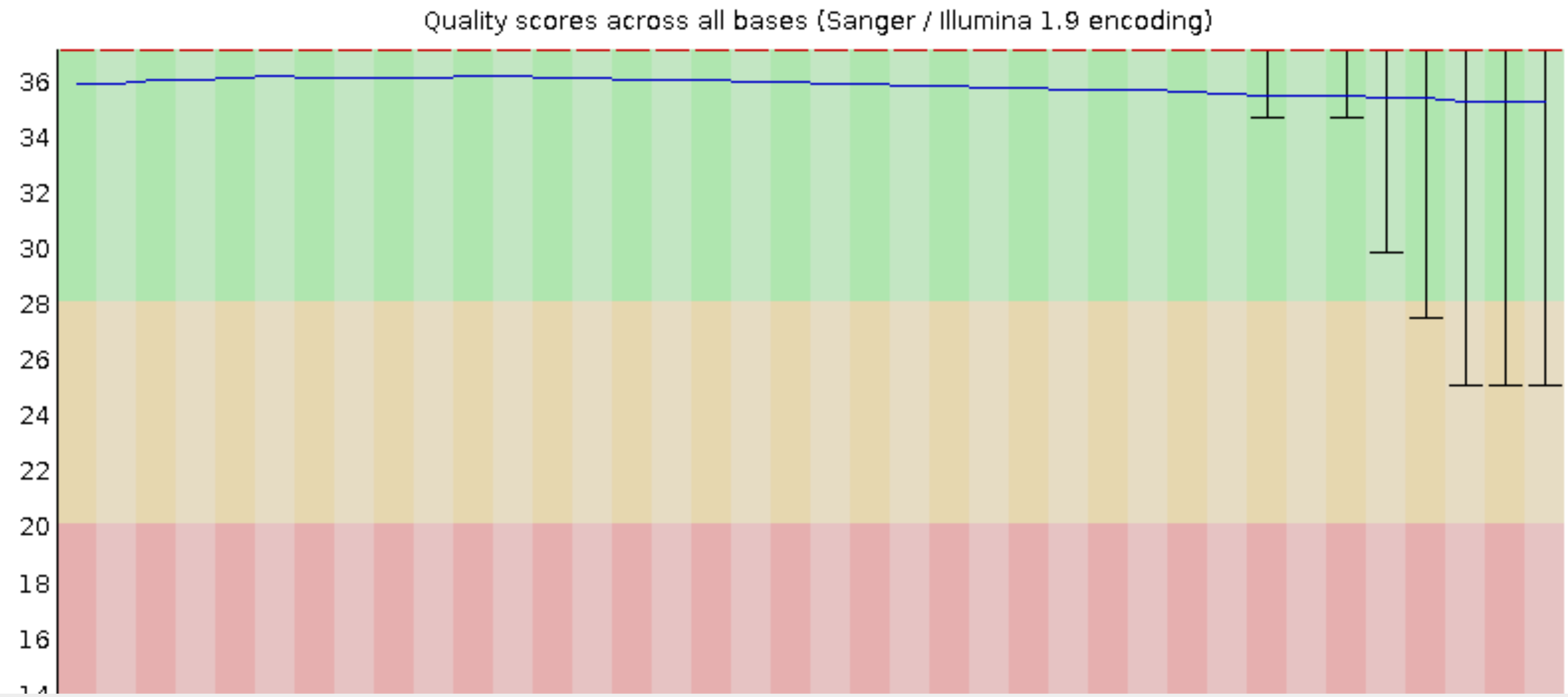

Supplement: Supplementary Link 1 — FastQC output html file (LR13_HHH23DSXX_L3_1_fastqc.html). [file Data_Sheet_6.zip › SupplementaryLink_2.pdf]
